# Relevance of GC content to the conservation of DNA polymerase III/mismatch repair system in Gram-positive bacteria

**DOI:** 10.3389/fmicb.2013.00266

**Published:** 2013-09-17

**Authors:** Motohiro Akashi, Hirofumi Yoshikawa

**Affiliations:** Department of Bioscience, Tokyo University of AgricultureTokyo, Japan

**Keywords:** DNA polymerase III, GC content, mismatch repair, Gram-positive, Actinobacteria

## Abstract

The mechanism of DNA replication is one of the driving forces of genome evolution. Bacterial DNA polymerase III, the primary complex of DNA replication, consists of PolC and DnaE. PolC is conserved in Gram-positive bacteria, especially in the Firmicutes with low GC content, whereas DnaE is widely conserved in most Gram-negative and Gram-positive bacteria. PolC contains two domains, the 3′-5′exonuclease domain and the polymerase domain, while DnaE only possesses the polymerase domain. Accordingly, DnaE does not have the proofreading function; in *Escherichia coli*, another enzyme DnaQ performs this function. In most bacteria, the fidelity of DNA replication is maintained by 3′-5′ exonuclease and a mismatch repair (MMR) system. However, we found that most Actinobacteria (a group of Gram-positive bacteria with high GC content) appear to have lost the MMR system and chromosomes may be replicated by DnaE-type DNA polymerase III with DnaQ-like 3′-5′ exonuclease. We tested the mutation bias of *Bacillus subtilis*, which belongs to the Firmicutes and found that the wild type strain is AT-biased while the *mutS*-deletant strain is remarkably GC-biased. If we presume that DnaE tends to make mistakes that increase GC content, these results can be explained by the *mutS* deletion (i.e., deletion of the MMR system). Thus, we propose that GC content is regulated by DNA polymerase and MMR system, and the absence of *polC* genes, which participate in the MMR system, may be the reason for the increase of GC content in Gram-positive bacteria such as Actinobacteria.

## Introduction

There are various external causes of genetic mutation in organisms, such as UV radiations, oxidative environment, or exposure to radiation. However, the main source of DNA mutation arises from replication errors caused by DNA polymerase in the organism itself. Although several DNA polymerases are conserved in the bacterial cell, the major DNA replication enzyme, DNA polymerase III, belongs to the Family C-type (McHenry, 2011). There are two types of the Family C-type replication enzymes: PolC, which conserves the proofreading apparatus of the 3′-5′ exonuclease domain in addition to the polymerase domain; and DnaE, which possesses the replication enzyme, α subunit. PolC and DnaE were originally Family A/B-type DNA polymerases but became Family C-type as the result of a mutation in the 3′-5′ exonuclease domain (Huang et al., 1997). Family C-type polymerase is currently considered PolC, whereas DnaE and DnaQ are derived from the separation of its two domains (Huang et al., 1997).

Mutations produced during DNA replication are restored by DNA repair enzymes or DNA replication proofreading enzymes, many of which are thought to be widely conserved by a domain unit, regardless of the species (Aravind et al., 1999). This fact supports the hypothesis that the α subunit and 3′-5′ exonuclease segregated during the evolutionary history of Family C-type DNA replication enzymes.

Change in the GC content of an organism is a useful index in biological classification, especially in prokaryotic classification. The bioinformatics investigation showed that change in the GC content and polymorphisms of bacterial PolC and DnaE are considered to have a co-evolutionary relationship (Wu et al., 2012). From the phylogenetic classification of several proteins, bacteria and archaea diverged around 4 billion years ago and further split into two large groups 2.5 billion years ago when oxygen concentrations on earth rose: one includes Proteobacteria and spirochaeta, the other Actinobacteria, cyanobacteria, and Firmicutes (Battistuzzi et al., 2004). This oxygen concentration increase supposedly caused an explosive differentiation of bacterial species. However, there are still many unanswered questions and too few concrete answers to explain the kind of events that occurred. Actinobacteria is one of the bacterial groups, which are considered to have diverged in this historical period of oxidization on earth. These bacteria possess a peptidoglycan layer and their genomes have a high GC content. On the other hand, *Bacillus subtilis* is Gram-positive and belongs to the Firmicutes with a genome of low GC content (43%) compared to Actinobacteria. An *in vitro* reconstitution experiment has shown that DnaE and PolC are essential in *B. subtilis* genome replication, with the former replicating the lagging strand while the latter replicating the leading strand (Dervyn et al., 2001; Le Chatelier et al., 2004).

*mutS* is a representative DNA mismatch repair (MMR) gene that is conserved among not only bacteria but also eukaryotes. In recent years, from studies of *B. subtilis*, it has been proposed that MutS binds to DnaN (the clamp of the DNA replication complex), repairs the mutation specific to the DNA non-methylated strand, and dissociates the DNA replication complex from DNA during the mutation repair of the errors during DNA replication (Lenhart et al., 2013).

We propose that the mutation factor is the interval between mutation causation by the DNA replication enzyme and restoration by the mutation repair enzyme. In this study, we validated the DNA replication enzyme conservation for several DNA repair enzymes, and we performed an *in vivo* mutational analysis by using *B. subtilis*.

## Materials and methods

### Phylogenetic classification of DNA polymerase III and mutS/L/T

To study the conservation of DNA polymerase III, MutS, MutL, and MutT in bacteria, we investigated the amino acid sequences of each gene *dnaE* (NP_414726), *mutS* (NP_417213), *mutL* (NP_418591), and *mutT* (NP_414641) in *Escherichia coli* by using DELTA-BLAST (Boratyn et al., 2012). The list of the investigated bacteria is shown in Supplemental Table 1. The acquired amino acid sequence of DNA polymerase III was analysed using MEGA5 (Tamura et al., 2011). All sequences were aligned by Clustal W. Gaps included in the alignment were deleted at the last alignment step. Subsequently, a phylogenetic tree was created by the neighbor-joining method based on the alignment file, from which probability was confirmed by the 500-time trial using the bootstrap method.

### Rifampicin mutagenesis assay using *B. subtilis*

Two *B. subtilis* strains, strain 168 *trpC2* [hereafter referred as the wild type (WT) strain] and strain 168 *trpC2 mutS*::spec^r^, were used for mutation analysis. A frozen stock of each strain was precultured on LB plates and cultured in the LB liquid medium with rotation (48 rpm) at 37°C, for 8 or 24 h. Each culture was diluted by 10^−5^ and 10^−6^ and plated on LB plates, while the non-diluted cultures of the Δ*mutS* strain and WT strain were plated on LB plates containing 5 μg/ml of rifampicin. Colony forming unit (CFU) was calculated according to the number of colonies after 24 h incubation. The rate of mutation was defined as rifampicin-resistant CFU on LB plates with rifampicin per CFU on LB plates. This test was performed three times for each strain.

### Point mutation analysis in the rpoB region I

For the point mutation analysis, the two strains described above (WT and 168 *trpC2 mutS*::spec^r^) were also used. Twenty colonies from the WT strain and 30 colonies from the Δ*mutS* strain acquired from the rifampicin mutagenesis assay were selected randomly and the sequence of the *rpoB* region I was confirmed. The primers for PCR amplification and sequence analysis were *rpoB* +1157 (5′-gctacttcttcaacctgctgc-3′) for the forward and *rpoB* +1673 rev (5′-gttaccttccctgtttcagggtc-3′) for the reverse. Sequence analysis was performed by MACROGEN JAPAN (http://www.macrogen-japan.co.jp/).

## Results

### Extensive loss of MutS/L in actinobacteria

To confirm the correlation between the conservation of the MMR enzyme MutL/S/T and that of the bacterial DNA replication enzyme DNA polymerase III, we verified the conservation of bacterial DNA polymerase III and MutL/S/T by using a BLAST search. The genome analysis for all bacterial species investigated (Supplemental Table 1) has been completed or is currently in progress.

It is noteworthy that neither MutS nor MutL, both of which are MMR enzymes, were detected in actinobacteria by BLAST search, whereas MutT, which decomposes 8-oxoguanine, was found not only in Actinobacteria but also many other bacteria. Furthermore, we could not detect MutS, MutL, or MutT in *Mycoplasma mobile* strain 163K and *Spiroplasma melliferum* strain KC3 (Table 1).

**Table 1:** **Number of DNA polymerase III and typical MMR genes in each bacterium**.

**Phylum**	**Species**	**PolC**	**DnaQ**	**DnaE**	**MutS**	**MutL**	**MutT**
Proteobacteria	*Rhodobacter sphaeroides* ATCC17025	0	1	1	1	1	9
	*Rhodospirillum rubrum* ATCC11170	0	1	1	1	1	9
	*Pseudomonas* sp. M47T1	0	1	2	1	1	14
	*Zymomonas mobilissub* sp. *mobilis* ATCC29191	0	1	1	1	1	2
	*Acetobacter tropicalis* NBRC 101654	0	1	1	1	1	8
	*Gluconobacter morbifer* G707	0	1	1	1	1	6
	*Rickettsia felis* URRWXCal2	0	2	1	1	1	13
	*Caulobacter* sp. AP07	0	1	2	1	1	16
	*Nitrobacter* sp. Nb-311A	0	1	2	1	1	9
	*Hyphomicrobium denitrificans* 1NES1	0	1	2	1	1	8
	*Nitrosomonas* sp. Is79A3	0	1	1	1	1	5
	*Neisseria wadsworthii* 9715	0	3	1	1	1	7
	*Chromobacter iumviolaceum* ATCC12472	0	5	1	1	1	15
	*Leptothrix cholodnii* SP-6	0	5	2	1	1	7
	*Burkholderia glumae* BGR1	1	3	2	1	1	12
	*Thiobacillus denitrificans* ATCC25259	0	3	2	1	1	7
	*Bdellovibriobacter iovorus* str. Tiberius	0	4	2	3	1	5
	*Myxococcus xanthus* DK1622	0	2	2	3	1	13
	*Desulfovibrio* sp. FW1012B	0	1	1	2	1	5
	*Desulfuromonas acetoxidans* DSM684	0	2	1	2	1	10
	*Stigmatella aurantiaca* DW4/3-1	0	5	4	7	2	24
	*Desulfobacter postgatei* 2ac9	0	4	1	1	1	5
	*Campylobacter coli* 1417	0	1	1	1	1	2
	*Xanthomonas albilineans* GPEPC73	0	2	1	1	1	10
	*Ectothiorhodospira* sp. PHS-1	0	2	1	1	1	8
	*Methylomonas methanica* MC09	0	4	2	1	1	8
	*Methylobacter tundripaludum* SV96	0	2	1	1	1	8
	*Azotobacter vinelandii* DJ	0	5	1	1	1	10
	*Escherichia coli* TA143	0	3	1	1	1	13
	*Salmonella enterica* subsp. *enterica* serovar Typhi str. CT18	0	5	2	1	1	13
	*Proteus penneri* ATCC 35198	0	2	2	2	1	13
	*Enterobacter cancerogenus* ATCC 35316	0	3	1	1	1	15
	*Vibrio cholerae* MZO-2	0	5	1	1	1	12
	*Photobacterium leiognathi* subsp. *mandapamensis* svers.1.1.	0	6	1	1	1	14
	*Beggiatoa alba* B18LD	0	5	1	1	1	12
	*Coxiella burnetii* Dugway 5J108-111	0	2	1	2	2	5
Actinobacteria	*Micrococcus luteus* SK58	0	3	2	0	0	9
	*Propionibacterium propionicum* F0230a	0	1	1	0	0	10
	*Streptomyces* sp. AA4	0	3	4	0	0	27
	*Actinomyces* sp. ICM39	0	2	2	0	0	6
	*Corynebacterium amycolatum* SK46	0	4	2	0	0	10
	*Arthrobacter* sp. FB24	0	3	2	0	0	15
	*Mycobacterium fortuitum* subsp. *fortuitum* DSM 46621	0	4	2	0	0	15
Firmicutes	*Staphylococcus capitis* SK14	1	2	1	2	1	7
	*Lactobacillus sakei* subsp. *sakei* 23K	1	2	1	2	1	4
	*Bacillus subtilis* subsp. *spizizenii* ATCC 6633	1	3	1	2	1	6
	*Sporosarcina newyorkensis* 2681	1	4	1	3	1	7
	*Clostridium thermocellum* ATCC 27405	1	1	1	3	1	5
	*Heliobacterium modesticaldum* Ice1	1	1	1	2	1	1
	*Streptococcus pneumoniae* SP14-BS69	1	3	2	2	2	8
	*Mycoplasma mobile* 163K	1	1	1	0	0	0
Tenericutes	*Spiroplasma melliferum* KC3	1	0	1	0	0	0
Cyanobacteria	*Synechococcus elongatus* PCC 7942	0	1	2	4	1	5
	*Oscillatoria* sp. PCC 6506 (*Oscillatoria* sp. PCC 9029)	0	2	1	1	1	11
	*Nostoc* sp. PCC 7107	0	2	2	2	1	9
Chlamydiae	*Chlamydia muridarum* Nigg	0	2	1	1	1	1
Planctomycetes	*Planctomyces limnophilus* DSM 3776	0	0	2	1	1	3
	*Pirellula staleyi* DSM 6068	0	2	1	1	0	5
Bacteroidetes	*Bacteroides* sp. 2 1 7	0	4	1	4	1	6
	*Cytophaga hutchinsonii* ATCC 33406	0	3	1	3	1	8
Chlorobi	*Chlorobium ferrooxidans* DSM 13031	0	2	1	2	1	6
	*Prosthecochloris aestuarii* DSM 271	0	4	1	2	1	7
Spirochaetes	*Spirochaeta africana* DSM 8902	0	1	2	3	1	4
	*Treponema pallidum* subsp. *pallidum* str. Nichols	0	1	1	1	2	1
Deinococcus-Thermus	*Deinococcus gobiensis* I-0	0	3	1	2	1	17
	*Thermus* sp. RL	0	3	1	2	1	6
Chloroflexi	*Chloroflexus aggregans* DSM 9485	0	3	1	2	1	11
	*Thermomicrobium roseum* DSM 5159	0	4	2	3	2	7
Thermotogae	*Thermotoga neapolitana* DSM 4359	1	1	1	2	1	2
Aquificae	*Aquifex aeolicus* VF5	0	3	1	2	1	2

### Strains with extremely biased GC content of DNA possess the same type of DnaE

**Figure 1:**
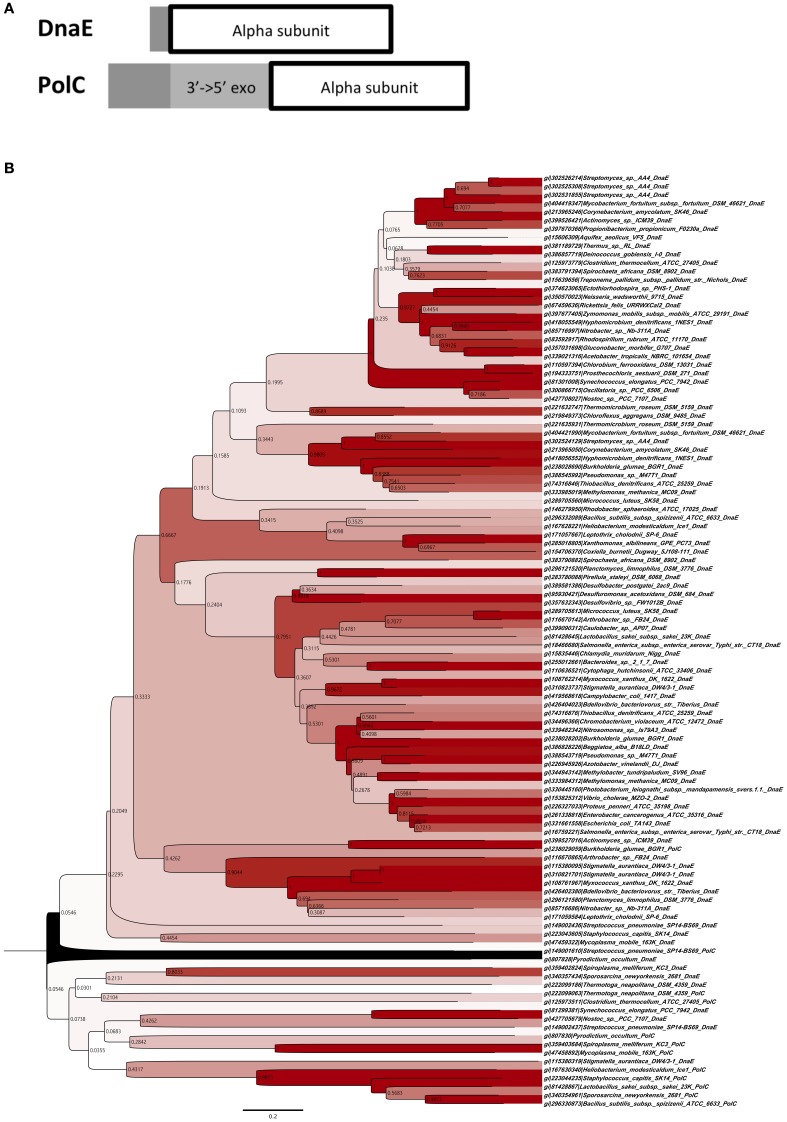
**Phylogenetic classification of DNA polymerase III. (A)** Domain construction of two different types of DNA polymerase III α subunits. “3′ → to 5′exo” indicates the 3′to 5′ exonuclease domain. “Alpha subunit” indicates the DNA polymerase III α subunit domain. **(B)** Phylogenetic tree of DNA polymerase III α subunits of DnaE and PolC, using sequences of 110 proteins with the neighbor-joining method. Bootstrap values are shown on each node. The contrast of color between blanch depends on bootstrap values, e.g., higher values are in darker red.

Because MutS/L is not conserved in Actinobacteria, we speculated that the increase in GC content is due to amino acid sequence differences in the DNA replication enzyme domain of these bacteria. The aligned amino acid sequence of the DNA polymerase III α subunit domain from all bacteria was analysed. The algorithm of Clustal W was used for alignment, and phylogenetic analysis was performed by the neighbor-joining method based on the results of the alignment data (Figures 1A,B). From the results of phylogenetic analysis, the phylum Deinococcus-Thermus, which is known to have as high a GC content as Actinobacteria, and *Clostridium thermocellum*, which shows GC content as low as 39%, were included in the DnaE clade along with Actinobacteria, suggesting a relationship between the type of DNA polymerase and the instability of GC content.

### The mutation frequency of the Δ*MutS* strain is high in *B. subtilis* 168 and induces mutational bias of base change(s) to AT-GC

**Figure 2:**
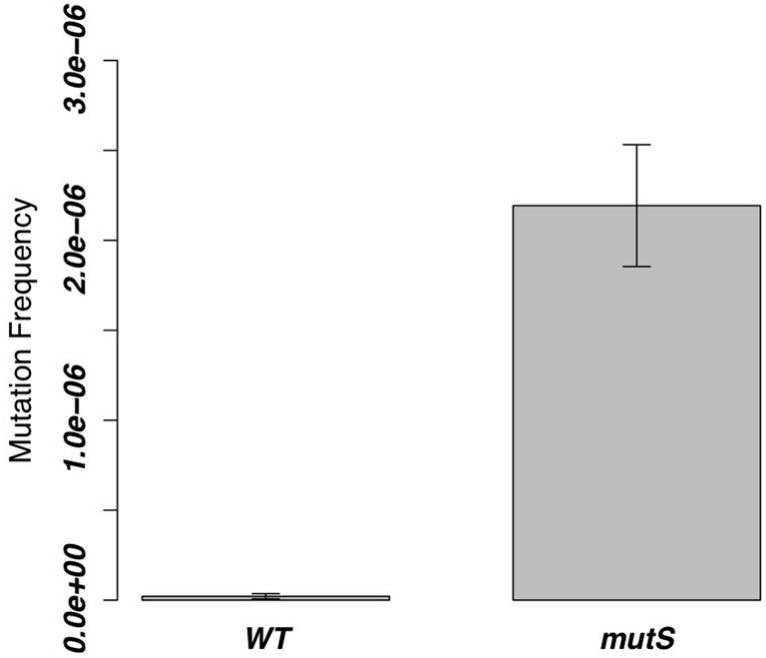
**Mutation frequencies of the Δ*mutS* strain in the 8 h-cultured fractions**. The same value of the WT strain was measured as a control, and the bar of each column indicates SD.

We deleted *mutS* of the Gram-positive *B. subtilis* 168 strain and measured the mutation frequency. As a result, the Δ*mutS* strain showed a 100-fold higher frequency of mutation than the WT strain (Figure 2).

**Figure 3:**
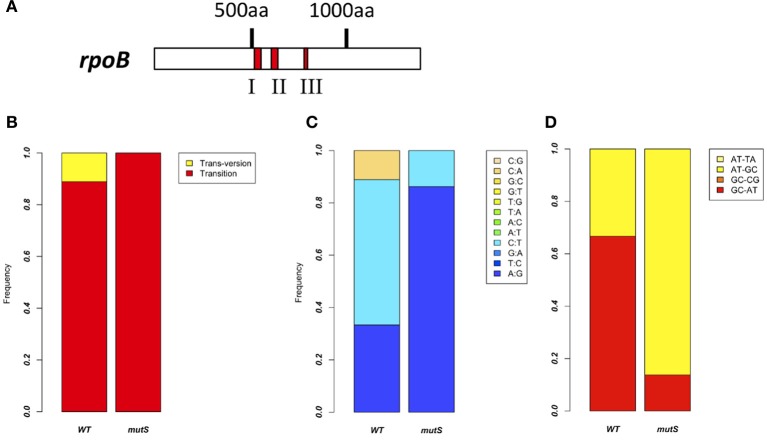
**Point mutation analysis in the *rpoB* region I**. **(A)** Rifampicin-resistant regions of the RNA polymerase ß Subunit. Red marks indicate the clusters where rifampicin-resistant mutations have been identified. Since highly conserved residues in these regions directly interact with rifampicin, we investigated region I of the *rpoB* gene. **(B)** Comparison of the *rpoB* mutation bias between the *B. subtilis* 168 WT strain and the Δ*mutS* strain focusing on mutation types. Red and yellow indicate transition mutation and trans-version mutation, respectively. **(C)** Comparison of *rpoB* mutation bias between the *B. subtilis* 168 WT strain and the Δ*mutS* strain focusing on a nucleotide change. For example, “A:G” means A to G change. **(D)** Comparison of *rpoB* mutation bias between the *B. subtilis* 168 WT strain and the Δ*mutS* strain focusing on the GC bias. “AT-TA” and “GC-CG” indicate the transversion mutation without change of the GC bias. “AT-GC” and “GC-AT” indicate the transition and transversion mutation changing the GC bias.

From the former experiment, we picked 20 rifampicin-resistant WT strains and 30 rifampicin-resistant Δ*mutS* strains. Since rifampicin is known to react on region I of the RNA polymerase β subunit gene (*rpoB*) (Campbell et al., 2001), we performed sequence analysis in this region to investigate the frequency of point mutations (Figure 3A). Ninety percent of mutations in the WT strain and all mutations in the Δ*mutS* strain were transition mutations (Figure 3B). The identification of these point mutations showed that the ratios of each base change were 11, 56, and 33% for the C-A, C-T, and A-G substitutions, respectively, in the WT strain, whereas they were 14% for the C-T substitution and 86% for A-G in the Δ*mutS* strain (Figure 3C). The classification of these mutations is as follows: GC content-increasing (AT-GC); GC content-decreasing (GC-AT); and GC content-unchanging (AT=TA, GC=CG). Accordingly, the AT-increasing (GC-AT) mutation in the WT strain was 67% of the total mutations. In contrast, the AT-GC mutation was 86% in the Δ*mutS* strain (Figure 3D). Therefore, it appears that the GC content increased as a result of the *mutS* deletion.

## Discussion

The mutation experiment results of the *B. subtilis* 168 strain shows that MutS suppresses the AT-GC mutation, based on evidence that the deletion of *mutS* induces mutational bias to AT-GC. In addition, many GC-AT mutations are observed in the *B. subtilis* 168 WT strain, suggesting the existence of other mechanisms that increase the AT content in this strain. MutS forms a complex with a clamp called DnaN during the DNA replication in *B. subtilis* (Lenhart et al., 2013). While PolC replicates the leading strand of *B. subtilis* and has a proofreading function, DnaE, which synthesizes the lagging strand, does not possess this function. If the MutS/L complex takes a central role in MMR of the lagging strand, it is possible that the mutation in the MutS-deleted strain is located mainly in the lagging strand. Simultaneously, DnaE as a replication enzyme tends to create the AT-GC mutation, while MutS represses this mutational bias.

Actinobacteria possess only the DnaE-type (that does not contain 3′-5′ exonuclease activity) DNA polymerase and does not conserve MutS/L. Furthermore, since Actinobacteria conserves MutT, it is not likely that the increase of 8-oxoguanine (8-OG) raises the GC content. Actinobacteria and Deinococcus, both with high GC content are thought to have branched about 2.5 billion years ago when the oxidation of the earth's atmosphere by cyanobacteria supposedly occurred (Battistuzzi et al., 2004). Many organisms existing at this time would have experienced this external oxidation event and those intolerant of this change would not have survived. It is difficult to directly correlate the mutation induced by 8-OG, which should occur in the organism growing in the aerobic environment with its increase in GC content. Basically, in the case of the cell in a constant environment, the main power to mutate DNA depends on the replication error rate of the DNA polymerase. Thus, the loss of PolC and MutS/L might induce the increase of GC content during the speciation of Actinobacteria.

Based on the results from the DNA polymerase classification, bacteria with extremely high or low GC contents such as Actinobacteria, Deinococcus-Thermus, and *Clostridium* were classified into the same clade (Figure 1B). Bacteria have experienced the appearance of both *polC* and *dnaE* genes during their evolutionary history. Assuming that these enzymes may originally have been the driving force pulling the GC content from both sides, this might suggest the existence of a balance between maintenance of the unstable state of DNA-composing bases and the type of their DNA polymerases. Although many bacteria have lost PolC, most have acquired the mutant with little change in GC content and evolved. Among these, the ancestor of Actinobacteria may have experienced the rise of its GC content after the loss of PolC.

We could not find MutS, MutL, or MutT in *M. mobile* 163K and *S. melliferum* KC3 by BLAST search. These bacteria belong to Tenericutes or Firmicutes and replicate DNA by using the PolC-type DNA polymerase. Since their GC contents are extremely low (23~40%), it has been suggested that the error bias of the DNA replication enzyme and lack of a mechanism to repress mutational errors has induced this extreme decrease. It remains unknown, however, how these bacteria maintain such low GC content, or the type of mechanism that exists to correct replication mistakes.

Since there is proximity between the DnaE-type DNA polymerases of thermophiles Deinococcus-Thermus and *C. thermocellum* and one species of Actinobacteria, there seems to be no relationship between the increase and decrease of GC content and the type of DNA polymerase. However, it is possible that DNA polymerase is associated with extreme increases or decreases of GC content. While increased GC content in Deinococcus-Thermus and Actinobacteria is due to a balance between DNA polymerase and repair, *C. thermocellum* possesses not only DnaE-type but also PolC-type DNA polymerases and the balance between these multiple polymerases and DNA repair lowered the GC content. Furthermore, together with the mutation analysis results of *B. subtilis* strain 168, which replicates DNA by PolC and DnaE and the deletion of its *mutS*, which increases the GC content, it has signified that MutS itself possesses the function to repress increasing GC content. Since the function of MutS is the recognition of the MMR, it is unlikely that it has the potency to self-introduce mutations; therefore, its function to repress increasing GC content is thought to be a passive reaction. We first thought that the change of GC content was the result of the fitting of DnaE to the genomic mutation, which occurs due to the loss of MutS; however, this does not explain the change of GC content in Deinococcus-Thermus and *C. thermocellum* while still maintaining MutS/L. Therefore, we suggest that the increase of the GC content in Actinobacteria is induced by the type of its DNA replication enzyme and the loss of MutL/S. However, this analogy does not apply to examples such as *M. mobile* 163K and *S. melliferum* KC3, perhaps because the conditions necessary for the proof have not been established.

### Conflict of interest statement

The authors declare that the research was conducted in the absence of any commercial or financial relationships that could be construed as a potential conflict of interest.

## References

[B1] Aravind L. Walker D. R. Koonin E. V. (1999). Conserved domains in DNA repair proteins and evolution of repair systems. Nucleic Acids Res. 27, 1223–1242 10.1093/nar/27.5.1223 9973609 PMC148307

[B2] Battistuzzi F. U. Feijao A. Hedges S. B. (2004). A genomic timescale of prokaryote evolution: insights into the origin of methanogenesis, phototrophy, and the colonization of land. BMC Evol. Biol. 4:44 10.1186/1471-2148-4-44 15535883 PMC533871

[B3] Boratyn G. M. Schäffer A. A. Agarwala R. Altschul S. F. Lipman D. J. Madden T. L. (2012). Domain enhanced lookup time accelerated BLAST. Biol. Direct 7:12 10.1186/1745-6150-7-12 22510480 PMC3438057

[B4] Campbell E. A. Korzheva N. Mustaev A. Murakami K. Nair S. Goldfarb A. (2001). Structural mechanism for rifampicin inhibition of bacterial RNA polymerase. Cell 104, 901–912 10.1016/S0092-8674(01)00286-0 11290327

[B5] Dervyn E. Suski C. Daniel R. Bruand C. Chapuis J. Errington J. (2001). Two essential DNA polymerases at the bacterial replication fork. Science 294, 1716–1719 10.1126/science.1066351 11721055

[B6] Huang Y. Braithwaite D. K. Ito J. (1997). Evolution of dnaQ, the gene encoding the editing 3′to 5′exonuclease subunit of DNA polymerase III holoenzyme in Gram-negative bacteria. FEBS Lett. 400, 94–98 10.1016/S0014-5793(96)01361-0 9000520

[B7] Le Chatelier E. Bécherel O. J. d'Alençon E. Canceill D. Ehrlich S. D. Fuchs R. P. P. (2004). Involvement of DnaE, the second replicative DNA polymerase from Bacillus subtilis, in DNA mutagenesis. J. Biol. Chem. 279, 1757–1767 10.1074/jbc.M310719200 14593098

[B8] Lenhart J. S. Sharma A. Hingorani M. M. Simmons L. A. (2013). DnaN clamp zones provide a platform for spatiotemporal coupling of mismatch detection to DNA replication. Mol. Microbiol. 87, 553–568 10.1111/mmi.12115 23228104 PMC5938748

[B9] McHenry C. S. (2011). Breaking the rules: bacteria that use several DNA polymerase IIIs. EMBO Rep. 12, 408–414 10.1038/embor.2011.51 21475246 PMC3090020

[B10] Tamura K. Peterson D. Peterson N. Stecher G. Nei M. Kumar S. (2011). MEGA5: molecular evolutionary genetics analysis using maximum likelihood, evolutionary distance, and maximum parsimony methods. Mol. Biol. Evol. 28, 2731–2719 10.1093/molbev/msr121 21546353 PMC3203626

[B11] Wu H. Zhang Z. Hu S. Yu J. (2012). On the molecular mechanism of GC content variation among eubacterial genomes. Biol. Direct 7:2 10.1186/1745-6150-7-2 22230424 PMC3274465

