# Supplementary material for: Relevance of GC content to the conservation of DNA polymerase III/mismatch repair system in Gram-positive bacteria
Source: Front Microbiol. 2013 Sep 17;4:266. doi: 10.3389/fmicb.2013.00266 (PMC3774996; doi:10.3389/fmicb.2013.00266)
Supplement: Supplementary file 1 [file DataSheet1.DOCX]

Supplemental Table 1

Bacterial species analysed in this study.

| phylum | class | genus | species and strain | Tax ID | GC (%) |
| --- | --- | --- | --- | --- | --- |
| Proteobacteria | Alpha-proteobacteria | *Rhodobacter* | *sphaeroides* ATCC17025 | 349102 | 68 |
|  |  | *Rhodospirillum* | *rubrum* ATCC11170 | 269796 | 65 |
|  |  | *Pseudomonas* | sp. M47T1 | 1179778 | 64 |
|  |  | *Zymomonas* | *mobilis* subsp*. mobilis* ATCC29191 | 627344 | 46 |
|  |  | *Acetobacter* | *tropicalis* NBRC101654 | 749388 | 55 |
|  |  | *Gluconobacter* | *morbifer* G707 | 1088869 | 59 |
|  |  | *Rickettsia* | *felis* URRWXCal2 | 315456 | 33 |
|  |  | *Caulobacter* | sp. AP07 | 1144304 | 67 |
|  |  | *Nitrobacter* | sp. Nb-311A | 314253 | 60 |
|  |  | *Hyphomicrobium* | *denitrificans* 1NES1 | 670307 | 60 |
|  | Beta-proteobacteria | *Nitrosomonas* | ssp. Is79A3 | 261292 | 45 |
|  |  | *Neisseria* | *wadsworthii* 9715 | 1030841 | 49 |
|  |  | *Chromobacterium* | *violaceum* ATCC12472 | 243365 | 65 |
|  |  | *Leptothrix* | *cholodnii* SP-6 | 395495 | 69 |
|  |  | *Burkholderia* | *glumae* BGR1 | 626418 | 68 |
|  |  | *Thiobacillus* | *denitrificans* ATCC25259 | 292415 | 66 |
|  | Delta-proteobacteria | *Bdellovibrio* | *bacteriovorus* str. *Tiberius* | 1069642 | 50 |
|  |  | *Myxococcus* | *xanthus* DK1622 | 246197 | 69 |
|  |  | *Desulfovibrio* | sp. FW1012B | 644968 | 67 |
|  |  | *Desulfuromonas* | *acetoxidans* DSM684 | 281689 | 52 |
|  |  | *Stigmatella* | *aurantiaca* DW4/3-1 | 378806 | 67 |
|  |  | *Desulfobacter* | *postgatei* 2ac9 | 879212 | 47 |
|  | Epsilon-proteobacteria | *Campylobacter* | *coli* 1417 | 887293 | 50 |
|  | Gamma-proteobacteria | *Xanthomonas* | *albilineans* GPEPC73 | 380358 | 63 |
|  |  | *Ectothiorhodospira* | sp. PHS-1 | 519989 | 64 |
|  |  | *Methylomonas* | *methanica* MC09 | 857087 | 51 |
|  |  | *Methylobacter* | *tundripaludum* SV96 | 697282 | 50 |
|  |  | *Azotobacter* | *vinelandii* DJ | 322710 | 66 |
|  |  | *Escherichia* | *coli* TA143 | 656437 | 51 |
|  |  | *Salmonella* | *enterica* subsp. *enterica* serovar *Typhi* str. CT18 | 220341 | 52 |
|  |  | *Proteus* | *penneri* ATCC35198 | 471881 | 38 |
|  |  | *Enterobacter* | *cancerogenus* ATCC35316 | 500639 | 56 |
|  |  | *Vibrio* | *cholerae* MZO-2 | 417398 | 48 |
|  |  | *Photobacterium* | *leiognathi* subsp. *mandapamensis* svers.1.1. | 1001530 | 41 |
|  |  | *Beggiatoa* | *alba* B18LD | 395493 | 41 |
|  |  | *Coxiella* | *burnetii* Dugway 5J108-111 | 434922 | 42 |
| Actinobacteria | Actinobacteria | *Micrococcus* | *luteus* SK58 | 596312 | 73 |
|  |  | *Propionibacterium* | *propionicum* F0230a | 767029 | 66 |
|  |  | *Streptomyces* | sp. AA4 | 591158 | 70 |
|  |  | *Actinomyces* | sp. ICM39 | 1105029 | 66 |
|  |  | *Corynebacterium* | *amycolatum* SK46 | 553204 | 59 |
|  |  | *Arthrobacter* | sp. FB24 | 290399 | 65 |
|  |  | *Mycobacterium* | *fortuitum* subsp. *fortuitum* DSM46621 | 1214102 | 66 |
| Firmicutes | Bacilli | *Staphylococcus* | *capitis* SK14 | 553212 | 33 |
|  |  | *Lactobacillus* | *sakei* subsp. *sakei* 23K | 314315 | 41 |
|  |  | *Bacillus* | *subtilis* subsp. *spizizenii* ATCC6633 | 703612 | 44 |
|  |  | *Heliobacterium* | *modesticaldum* Ice1 | 498761 | 57 |
|  | Clostridia | *Sporosarcina* | *newyorkensis* 2681 | 1027292 | 42 |
|  |  | *Clostridium* | *thermocellum* ATCC27405 | 203119 | 39 |
|  | Coccus | *Streptococcus* | *pneumoniae* SP14-BS69 | 406560 | 40 |
|  | Mollicutes | *Mycoplasma* | *mobile* 163K | 267748 | 25 |
| Tenericutes | Mollicutes | *Spiroplasma* | *melliferum* KC3 | 570509 | 27 |
| Cyanobacteria | Cyanobacteria | *Synechococcus* | *elongatus* PCC7942 | 1140 | 55 |
|  | Cyanophyceae | *Oscillatoria* | sp. PCC6506 (sp. PCC9029) | 272129 | 43 |
|  |  | *Nostoc* | sp. PCC7107 | 317936 | 40 |
| Chlamydiae | Chlamydiae | *Chlamydia* | *muridarum* Nigg | 243161 | 40 |
| Planctomycetes | Planctomycetia | *Planctomyces* | *limnophilus* DSM3776 | 521674 | 54 |
|  |  | *Pirella* | *staleyi* DSM6068 | 530564 | 57 |
| Bacteroidetes | Bacteroidia | *Bacteroides* | sp. 2 1 7 | 457388 | 45 |
|  | Cytophagia | *Cytophaga* | *hutchinsonii* ATCC33406 | 269798 | 39 |
| Chlorobi | Chlorobia | *Chlorobium* | *ferrooxidans* DSM13031 | 377431 | 50 |
|  |  | *Prosthecochloris* | *aestuarii* DSM271 | 290512 | 50 |
| Spirochaetes | Spirochaetia | *Spirochaeta* | *africana* DSM8902 | 889378 | 58 |
|  |  | *Treponema* | *pallidum* subsp. *pallidum* str. *Nichols* | 243276 | 53 |
| Deinococcus | Deinococci | *Deinococcus* | *gobiensis* I-0 | 745776 | 69 |
| -Thermus |  | *Thermus* | sp. RL | 456163 | 69 |
| Chloroflexi | Chloroflexi | *Chloroflexus* | *aggregans* DSM9485 | 326427 | 56 |
|  | Thermomicrobia | *Thermomicrobium* | *roseum* DSM5159 | 309801 | 64 |
| Thermotogae | Thermotogae | *Thermotoga* | *neapolitana* DSM4359 | 309803 | 47 |
| Aquificae | Aquificae | *Aquifex* | *aeolicus* VF5 | 224324 | 43 |

We studied 14 phyla, 24 classes, and 70 genera and species. The word ‘tax ID’ indicates the NCBI taxonomy accession number of genera and species, respectively.

This classification is based on the ′Taxonomy′ of NCBI (http://www.ncbi.nlm.nih.gov/taxonomy/).
